# It’s about living a normal life: parents’ quality of life when their child has a life-threatening or life-limiting condition - a qualitative study

**DOI:** 10.1186/s12904-024-01417-3

**Published:** 2024-04-09

**Authors:** Trine Brun Kittelsen, Vibeke Bruun Lorentsen, Charlotte Castor, Anja Lee, Lisbeth Gravdal Kvarme, Anette Winger

**Affiliations:** 1 Faculty of Health Sciences, Department of Nursing and Health Promotion, Oslo Metropolitan University, Pilestredet 32, Oslo, 0167 Norway; 2 Faculty of Health Sciences, Institute for Nursing, VID Specialized University, Oslo, Norway; 3 Department of Health Sciences, Lund University, Lund, Sweden; 4 Division of Pediatric and Adolescent Medicine, Oslo University Hospital HF, Oslo, Norway

**Keywords:** Quality of life, Parents, Children, Life-threatening, Life-limiting, Pediatric palliative care

## Abstract

**Background:**

Pediatric palliative care (PPC) seeks to enhance the quality of life (QoL) for both children and their families. While most studies within PPC have focused on the ill child’s QoL, less is known about parents’ experiences of their own QoL. The aim of this study was to explore parents’ QoL when their child has a life-threatening or life-limiting condition.

**Methods:**

The study has a qualitative, hermeneutic phenomenological design inspired by van Manen’s phenomenology of practice. In-depth interviews were conducted with 12 fathers and 12 mothers of children living with cancer or a genetic condition. A deeper understanding of parents’ lived experiences was obtained through an adapted photo elicitation method. Two rounds of thematic analysis were conducted, covering both the photo elicitation data, and interview data.

**Results:**

The findings describe four themes related to parents’ QoL: living a normal life, giving my child a good life, having time to fulfill siblings’ needs, and feeling heard and respected in the health and social care system.

**Conclusions:**

The complexity of elements shaping parents’ QoL is evident. The interconnectedness between parents, the ill child, siblings, and interactions with the health and social care system, highlights the need to understand and address diverse aspects in enhancing parents QoL.

**Supplementary Information:**

The online version contains supplementary material available at 10.1186/s12904-024-01417-3.

## Background

Pediatric palliative care (PPC) encompasses the care of children with life-threatening or life-limiting (LT/LL) conditions, regardless of their diagnosis or stage of illness [1]. The World Health Organization estimates that 21 million children worldwide live with LT/LL conditions [2]. Families with children in need of PPC encounter a range of challenges, including stress on siblings, physical and mental health issues, financial and work problems, social isolation, distress [3, 4], and changes in family relationships [5]. Parents’ support needs are substantial and heterogeneous [3] as they face many complex and challenging decisions [6] for which they need to constantly adapt, adjust, and reevaluate the child’s health status and their parenting actions and goals [5]. One study showed that parents spend an average of nine hours a day providing palliative care for children at home [7]. The provision of complex care, such as tube feeding, tracheostomy, mechanical ventilation symptom management, and support for their other children, may result in parents experiencing physical exhaustion and fatigue [8–10]. Despite parents reporting positive appraisals of the caregiving role [11], they also report feeling frustrated and hopeless, having cognitive problems remembering and focusing on tasks, and being anxious about the child’s future [12]. Research indicates that half of the parents providing care for a child with a life-limiting disease may fulfill the criteria for one or more clinically elevated diagnoses of stress, anxiety, or depression throughout the caregiving period [11]. In this situation, parents’ QoL may be threatened.

PPC seeks to enhance the quality of life (QoL) for both children and their families [2, 13]. QoL is internationally recognized as an outcome measure in PPC [9, 14]. While most studies within PPC have focused on the ill child’s QoL, less is known about the QoL of parents, who are often asked to report on their children’s QoL rather than their own [15–17]. QoL is a complex and broad concept that can be defined in different ways [18]. Generally, QoL is described as a multidimensional and subjective measure in health care [19]. The WHO defines QoL as an individual’s perceptions of their position in life in the context of the culture and value systems in which they live and in relation to their goals, expectations, standards, and concerns [20]. In more operational terms, QoL is an individual’s perceptions of their own functioning and well-being in different domains of life [20], such as health-related QoL, which narrows QoL to health-related concerns [21].

Some quantitative studies have approached parents’ QoL through measurement tools such as World Health Organization Quality of Life (WHOQOL) [22], PedsQL [12] or the FACT-General Population [11]. Other previous studies on parents’ experiences have primarily focused on parental coping, adjustment, and unmet needs [23–25]. Still, there remains a paucity of qualitative research exploring the QoL of parents of children with LT/LL conditions. Given that PPC aims to promote QoL for the whole family, more research is needed to better understand what QoL means for parents of children with LT/LL conditions. This study applied hermeneutic phenomenology to better understand parents’ lived experiences of their own QoL. The aim of this study was to explore parents’ QoL when their child has a life-threatening or life-limiting condition.

## Methods

### Design

In this study, we used a qualitative, hermeneutic phenomenological design inspired by van Manen’s phenomenology of practice [26] to investigate parents’ QoL when their child has a LT/LL condition. Phenomenology of practice is a context-sensitive form of descriptive and interpretive inquiry in which knowledge about the phenomenon itself can lead to more sensitive and caring professional services. A phenomenological descriptive sensitivity is combined with an interpretive understanding of lived experiences and how they are given meaning [27]. The research process was guided by van Manen’s lifeworld existentials – lived time, lived body, lived space, and lived relation – which are described as the fundamental structure of every person’s lifeworld [26]. Further, the philosophy of PPC serves as the frame of reference for this study [28]. This article adheres to the consolidated criteria for reporting qualitative research (COREQ) [29].

### Recruitment and participants

This study was part of a comprehensive study about the whole family’s (children, siblings, and parents) experiences of living with LT/LL conditions. For this paper, we focus on parents’ experiences. We recruited families in two ways: through three hospitals in the southeastern health region of Norway and three user organizations represented in the research network Children in Palliative Care [30]. Contact persons working at the hospitals recruited families by directly approaching parents and asking if they wanted to participate. User organizations informed parents about the study in meetings, a closed Facebook group, and on a webpage. Families who were interested in participating provided consent to be contacted by the first author, with the exception of two families who initiated direct contact with the first author themselves. One family declined to participate after receiving more information. As the study aimed at exploring the overarching phenomenon of living with a child with a LT/LL condition, we recruited families regardless of the ill child’s diagnosis. Families were included based on the following inclusion criteria, regardless of their language or ethnicity: (a) their child was under the age of 18, (b) their child had an LT or LL condition, (c) their child was under ongoing care, not recently diagnosed or in the end-of-life phase, and (d) the child lived primarily at home.

We recruited 24 parents (12 fathers and 12 mothers, all cohabitating) from September 2021 to March 2022. All parents spoke Norwegian. In two families, the ill child was the only child. Three children were diagnosed with cancer (category 1) at the age of 2–3, and two had suffered relapses. Nine children had genetic disorders (categories 3 and 4), three of whom were diagnosed in the first year of life and six of whom were diagnosed later (Table 1). The children in categories 3 and 4 had extensive medical complexity, with permanent cognitive and motor impairment. The authors did not have any prior knowledge of the parents or their families.

**Table 1:** Demographic characteristics of parents (*N* = 24) and their ill child (*N* = 12)

Characteristics	n
Parents’ age	
Mothers	27–53 (median 38)
Fathers	32–58 (median 40)
Parents’ education level	
Upper secondary school	11
Bachelor’s degree	8
Graduate studies	5
Ill child age (years)	1–17 (median 5)
LL/LT categories	
Category 1	3 (cancer)
Category 2	0
Category 3	8 (genetic)
Category 4	1 (genetic)
Category 5	0

a. Categories of LL/LT conditions: (1) Life-threatening conditions for which curative treatment may be feasible but can fail, (2) Conditions in which premature death is inevitable, (3) Progressive conditions without curative treatment options, (4) Irreversible but non-progressive conditions causing severe disability leading to susceptibility to health complications and the likelihood of premature death, (5) Unborn children who may not live through birth, infants who may survive for a few hours/days, infants with birth anomalies or for whom intensive care has been appropriately applied but developed an incurable disease (1)

### Data collection

The first author conducted in-depth interviews in the parents’ homes between October 2021 and April 2022. A pilot interview was conducted with parents, three siblings, and an ill child with an LL condition and highlighted the importance of allowing parents to freely express their experiences without the child or siblings present.

Ahead of interviews, parents provided information about their age and education level. Parents were generally interviewed together, except for two couples, who, for practical reasons, were interviewed separately in parts of the interview. In one interview, the ill child was sleeping in the same room, and in two other interviews, a sibling and the ill child were present for shorter periods. The parents were informed that the study aimed to explore their experiences of living with a child with an LT/LL condition, focusing on QoL. During the interview, the researcher aimed to foster an open-minded and empathetic atmosphere for parents to understand that there were no right or wrong answers. Parents were first asked to talk freely about their child and their situation, and during the interview, follow-up questions were asked to gain deeper insight into the parents’ experiences. All parents except the first couple were asked directly about their thoughts about QoL. The parents were also asked to talk about a good day and a bad day (Supplementary file 1). In a self-modified form of photo elicitation [31], parents brought objects and pictures that supported them in giving more in-depth descriptions of living with their ill child (Table 2). Ten of the families brought 16 objects and pictures. Both fathers and mothers were actively encouraged and supported to share their experiences during the interview. The interviews lasted 90 to 130 min and were recorded digitally, transcribed verbatim (with evidence of emotions marked in the transcript), and de-identified for analysis so that only the first author knew the participants’ identities. The first author called the parents by phone one to two weeks after the interview to ask about their well-being. Although three parents had found it emotionally challenging to participate in the interview, they all reported that it had been good to talk about their situation.

**Table 2:** List of objects and pictures parents brought to the interview

A birthday crown from the 17-year-old child’s first birthday A memory book from the time the ill child spent at the neonatal intensive care unit A toy skeleton of a human being that hung in the child’s room A birthday card drawn by the ill child A photo album of activities the family had done together A photo of the child smiling A slide in the ill child’s playroom A teddy bear given by the sibling brother to the ill child A drawing of the ill child at the hospital made by the sibling sister A spy bag that parents always brought along A coffee brewer brought to the hospital A swimming cap from the sibling sister A book about love language A picture of the playground outside the family’s house A picture of the family on top of a rock A picture of the family at the zoo	

### Analysis

Interviews were analyzed with a specific focus on answering the question: What is QoL for parents of children with LT/LL conditions? The analysis was conducted by the first author in collaboration with AW and VBL. Throughout the process, there was continuous discussion about the relationship between the parts and the whole. The analysis was conducted in two separate rounds, both guided by van Manen’s approach of systematic and discovery-oriented exploration of the phenomenon under study [27]. Prior to the two rounds of analysis, the interviews were read as open-mindedly as possible to get an overall sense of the material. Initial impressions were written down in an analysis document.

During the first round of analysis, we performed a thematic analysis of the objects and pictures presented by the parents during the interviews. In van Manen’s approach, thematic analysis is an open process of recovering the structures of meanings [27]. We reflected on the stories related to the objects/pictures by discussing their meaning in light of van Manen’s life existentials (lived time, lived space, lived body, and lived relation). All four life existentials were present in the interviews, but lived time was especially salient in the complexity of the parents’ experiences across the material. The objects and pictures were structured in meanings belonging together, and in this part of the analysis, we identified that parents’ lived experiences were distinctly connected to three broad preliminary themes (Table 3). The themes and reflections on the objects and pictures were added to the analysis document.

During the second round of analysis, we performed a thematic analysis of the interview material. To minimize overinterpretation in the first part of this analysis, we began by reading the sections in which parents explicitly described their perceptions of QoL. The three preliminary themes from the first round were distinctly present (Table 3). Additionally, a fourth preliminary theme not described through the objects and pictures emerged, so we incorporated it into the existing preliminary themes. We then proceeded by analyzing the rest of the interview material based on the four broad themes, searching for thicker descriptions and variations in the experiences that could provide a deeper understanding of parents’ QoL. The interviews were read repeatedly and thoroughly using van Manen’s selective reading approach. Phrases or statements that stood out and addressed aspects of QoL were added to the analysis document. The analysis document was reread several times and reflected upon through the lens of Manen’s life existentials, with special attention given to the parents’ choice of words to describe their situation.

During the whole process, analysis involved writing and rewriting to gain insight into the material [27], starting from reflection notes on parents’ concrete lived experiences and continuing until the organization of the written content revolving around the four themes. Quotes with fictitious names were chosen to highlight the meaning of the written results and the authors explored different titles to capture the essence and meaning of each theme. Finally, the first impressions recorded during the transcriptions were revisited, revealing that the immediate impressions were consistent with the findings in the written results.

**Table 3:** Analysis process

Preliminary Broad Themes	First Round Example of Object/Picture	Second Round Examples of Quotes Related to QoL	Life Existentials	Final Themes
Parents	*A picture of the playground outside the family’s house* The family went to the playground to get a sense of normal life in a strained situation (Family 3).	“Essentially, it’s about living a normal life. Quality of life comes when we can engage in ordinary activities” (Father, Family 4).	Time Body	Living a normal life
Ill child	*A toy skeleton of a human being that hung in the child’s room* The parents emphasized the toys’ significance in bringing joy to their child in various situations (Family 1).	“To me, quality of life means that Monica enjoys the best possible life. A good day for Monica is when she is free from pain and seizures. You can see the joy on her face, perhaps you even see a smile. Our ongoing goal is to ensure that she has pleasant days, getting to experience things and simply be a child at her age” (Father, Family 9).	Time Relation	Giving my child a good life
Siblings	*A picture of the ill child and the sibling at the zoo* Parents described the importance of *doing* things and letting the siblings have experiences while still being a child (Family 5).	“On the day he passes away, we don’t want his siblings to feel like their lives have been put on hold. It burdens us when they cannot engage in activities because we are unable to accompany them due to our focus on Morten” (Mother, Family 2).	Time Relation	Fulfilling siblings’ needs
System	Not addressed in this round of the analysis.	“You feel a profound sense of powerlessness. Because it doesn’t matter what you say or what you deliver in documentation of his needs. And it doesn’t even help what the physician in the specialist health service writes. You are told, indirectly, that you must pull yourself together. They have such enormous power. To decide our quality of life” (Mother, Family 6).	Relation Body	Feeling heard and respected by the health and social care system

### Preunderstanding

As researchers, our preunderstanding was based on our professional experience in nursing, occupational therapy, and clinical medicine at different levels in the healthcare system. We have worked with various diagnoses, phases of illness, and qualitative research within PPC. The research group consisted of women who were also mothers.

### Ethics

Ethical approval was obtained from the Regional Committees for Medical and Health Research Ethics in Norway (reference number 251,284), the Norwegian Center for Research Data (reference number 289,184), and the research ethics boards at each hospital involved in recruitment. Informed consent was obtained from all participants.

## Results

The findings are structured around four themes that describe parents QoL when their child has a LT/LL condition: living a normal life, giving my child a good life, fulfilling siblings’ needs, and feeling heard and respected by the health and social care system. The parents’ QoL was experienced as having a sense of normality, focusing on meaningful daily activities rather than letting the illness control their family life. Parents’ lived experiences also showed that their QoL was closely tied to how they perceived the well-being of their ill child and the siblings. This reflects the interconnected life worlds of the ill child, siblings, and parents themselves. Additionally, the health and social care system significantly impacted the parents’ QoL with how professionals communicated and offered services.

### Living a normal life

The parents emphasized that QoL was about living as normally as possible in a stressful and unpredictable life situation. Normality revolved around establishing a structured framework for each day, such as the ill child participating in kindergarten, school, or homeschooling. Rather than the absence of illness, normality was the ability to engage in meaningful everyday activities and routines that provided structure to the family’s daily life. Simple routines like preparing a packed lunch not only provided a sense of purpose but also served as a counterbalance to the stagnation and illness permeating the parents’ lives. “Quality of life is to do something **ordinary**. To be allowed to go to work. Have colleagues. Come home again. Just be a part of a normal world—the little things. To have something to hold on to in a surreal situation” (Father of a child with a genetic condition, Family 11).

For many parents, the opportunity to work played an important role in enhancing their QoL. Work was considered fulfilling, offering the opportunity to interact with others, spend time outdoors, enjoy solitude, and focus on things beyond the illness. While working contributed positively to their QoL, it also triggered existential quandaries concerning parents’ priorities. Questions like “Is it right to work and not prioritize my child?” and “Will I regret choosing to work?” fueled parents’ fears about spending their days on the right things, as the child’s life could be limited in time.

Being in the familiar home environment allowed parents to preserve a sense of normality and cherish positive moments within their daily routines instead of putting their lives on hold within the confines of a hospital. “Hospital at home” services, facilitating treatment and assessments in the comfort of their own environment, significantly contributed to the overall well-being and QoL of the parents.

The parents articulated the active creation of normality and QoL by carving out time for small pauses or breaks within their demanding and unpredictable circumstances. One couple illustrated this by bringing a coffee brewer to the interview. Reflecting on their experience, one parent stated, “I think it was the third day at the hospital, and we said that we would not sit there and drink lousy coffee in a situation with a child with cancer. So, I bought this coffee brewer that we brought along. One thing is that it gives us good coffee. It also represents our biggest strength as a couple, that we can find a small way to make things easier” (Mother of a child with cancer, Family 3).

### Giving my ill child a good life

Parents’ QoL was closely connected with their ill child’s well-being. When the ill child was doing well, the parents’ QoL improved. This enhancement was not solely attributed to the child’s well-being but also to the parents’ dedicated efforts to ensure a fulfilling life for their child. Parents’ beliefs about a good life for their child related to aspects such as the child being free from pain, spending time with family, and doing activities typical for the child’s age despite their illness or cognitive capacity; therefore, parents dedicated themselves to fulfilling these aspirations. One mother of a child with a genetic condition illustrated this commitment by showing a slide in the ill child’s playroom (Family 4). The slide demonstrated the ability to offer her son experiences typical for a five-year-old, despite the profound medical challenges he faced. This achievement, witnessed through her child’s enriched life, became a conduit for an enhanced parental QoL. The uncertainty about the length of the child’s life and the practical limitations of the situation prompted parents to adopt an attitude anchored in the present by finding joy in modest yet significant moments. This intentional focus on quality over quantity in life constituted an elevation of both the child’s and the parents’ QoL. “We know that he will have a short life. But we choose to focus on the quality of life instead of the length of life” (Mother of a child with a genetic condition, Family 2).

For parents, the child’s smile had a special meaning, as it confirmed the child’s well-being and supported parental QoL, especially when the child lacked verbal language. The smile provided a sense of fulfillment in the parents’ caregiving roles, thereby elevating their own QoL. Accordingly, the loss of the smile due to the severity of the illness impacted the parents’ QoL negatively. “When Maria started losing skills, the hardest part was losing her smile […] The smile has been there all along, which has been so fantastic. The smile was the sum of all her joy” (Mother of a child with a genetic condition, Family 8).

The pursuit of granting their child a good life was a double-edged sword. While it enhanced parental QoL, it also introduced a weighty responsibility, at times overwhelming, as some parents grappled with a sense of guilt for not constantly providing joyful experiences or being physically present at all times, even during brief intervals necessitated by daily tasks. A difficult balance emerged wherein the pursuit of a good life for the child, while improving their QoL, simultaneously engendered a strain that could weigh heavily on the parents. It was an intricate dance between responsibility and guilt in the interplay of fulfillment and burdensome obligation, which affected parents’ QoL.

### Fulfilling sibling needs

Parents’ QoL was negatively affected when they experienced challenges in fulfilling siblings’ needs because they were providing extensive care for the ill child. Due to the ill child’s comprehensive needs, there was often insufficient time to take care of the siblings as well, and this struggle led to a profound emotional impact characterized by a heightened sense of guilt, a burdened conscience, and an overarching feeling of inadequacy in their roles as parents. Parents worried that siblings were deprived of valuable experiences, were delayed in acquiring developmental skills such as swimming or bicycling, or were deprived of the possibility of going on holidays before they grew too old. “We often experience guilt, for example, when we’ve promised the brother a movie night. Sometimes unforeseen events happen with his ill brother, and time slips away. He’s out there with his popcorn bowl, waiting since quarter past eight to watch that movie with us. It’s not enjoyable. Not enjoyable at all” (Father of a child with a genetic condition, Family 9).

In balancing the parenting of both the ill child and siblings, parents sometimes needed practical help, such as respite care, to be able to attend to siblings, like accompanying siblings to recreational activities or assisting them with schoolwork. “Quality of life is having enough help so we can be a family and live, not just exist. All of us. It is not only about her” (Mother of a child with a genetic condition, Family 9). Opportunities to take care of siblings as well as the ill child improved parents’ QoL, and one couple illustrated this by bringing a bathing hat to the interview. This object symbolized that they could enjoy time with siblings in the swimming hall because they had assistance for the ill child at home (Parents of a child with a genetic condition, Family 5).

### Feeling heard and respected by the health and social care system

Parents reported a negative impact on their QoL when they experienced not being acknowledged or believed in encounters with healthcare or social system providers. One type of encounter that reduced QoL was with health care professionals (HCPs) in hospital settings. Despite parents’ expertise in understanding their child’s needs and nonverbal cues, they often felt dismissed as overprotective or demanding, with their competence overruled, especially when the healthcare provider was unfamiliar with the child or the child lacked a diagnosis. “When we do not have to fight in the system, and things work, I would say we have a good quality of life” (Father of a child with a genetic condition, Family 8).

Another challenging encounter that negatively affected parents’ QoL was with case managers from municipal service offices, where parents felt that the case manager lacked the knowledge to grasp the complexities of living with a child with an LT/LL condition. Parents who applied for services such as home assistants or respite care felt that they were perceived as a problem and an expense, and several parents talked about the feeling of standing with “hat in hand,” begging for help. “It is frightening that the municipality has so little knowledge of what it means to live in this situation. They do not have a clue what we are doing or how it affects us and the rest of the family” (Mother of a child with a genetic condition, Family 6).

When parents felt unheard or disbelieved, they felt forced to adopt a more assertive communication style to make their voices heard by avoiding displays of weakness and sometimes expressing frustration and anger. This shift had a negative impact on their QoL, as it was unpleasant and energy-draining to be this kind of parent. The gap between the severity of their situation and the caseworkers’ attempts to normalize it, for example, by saying that most families have night vigils when their children are young, exacerbated the problem, leaving parents feeling misunderstood and unsupported, and having their QoL negatively influenced. Some actions had the potential to lower the sense of powerlessness resulting from these situations, such as apologies from HCPs, physicians documenting the importance of listening to parents in medical records, or HCPs acting as “translators” between the hospital and the municipality to convey the gravity of their child’s condition.

## Discussion

The aim of this study was to explore parents’ QoL when their child has a LT/LL condition. Findings show that parents perceived QoL as living as normally as possible within the given circumstances, providing a good life for their ill child, meeting the needs of the siblings, and being supported by positive interactions with professionals in the health and social system. In this discussion, we elaborate on each of these four aspects of parental QoL.

Our study showed that parents experienced an improvement in their QoL when they found a sense of normality in their daily lives. Normality is a recurrent wish voiced by parents in other studies [32, 33], as it serves as a coping mechanism to lessen the impact of the ill child’s condition [5]. In our study, meaningful daily activities, such as the ill child attending school or kindergarten, served as a foundation for creating positive days within the parents’ challenging and unpredictable situation. In a meta-ethnographic exploration, Beecham [34] discovered that a crucial component of QoL for children with brain tumors and their families was the establishment of a “new normal” adapted to the family’s changed circumstances. This new normal was not a static measure but an ongoing repeated achievement through actions in daily life [34]. The parents in our study described normality as more achievable when they were at home than in the hospital, in line with various other studies [35–38]. According to Coombes’ study [39], achieving a sense of normality for parents requires time, space, and practical support but is often hindered by a lack of accessible assistance in practical, psychological, educational, and respite domains [39]. The findings suggest that HCPs in PPC should support parents in their struggle to maintain a new normality in their challenging situation, as this contributes to their overall well-being and QoL. Providing home-based PPC, encouraging respite care, or offering practical assistance can empower parents to participate in activities that foster a sense of normalcy in their lives.

Our study found that there was a close relationship between parents’ QoL and their perceptions of their ill child’s well-being. Existing research suggests that parents often rate their child’s QoL lower than the children themselves [40, 41]. Other studies have shown that a decrease in the child’s well-being corresponds to increased parental stress levels and a deterioration in their QoL [42, 43]. Further, the well-being of parents has a substantial impact on the well-being of their children [44, 45]. This poses a potential risk for a negative spiral in which parents consider their child’s QoL to be lower than it is, leading to a decline in the parents’ own QoL, subsequently impacting the ill child negatively. Recognizing and addressing these dynamics is crucial for providing comprehensive support to both parents and children within PPC, for example, by providing parents with education about assessing and understanding their child’s QoL more accurately.

Parents’ QoL was not only influenced by the ill child’s well-being but also by their own actions that affected the child’s QoL. This suggests that a sense of agency and commitment to their ill child’s well-being shaped the parents’ experience of their own QoL. Various studies [46–48] have shown that parents of ill children have deeply personal “good parents beliefs” [32], an ethical and weighty internal compass with which they often view themselves as duty-bound to act [47]. Previous research has shown that supporting parents in reaching their goal of being “good parents” can improve parental QoL [47]. Our study supports this finding and shows that parents facilitated a good life for their ill child by ensuring the child’s freedom from pain, inclusion in family activities, and engagement in age-appropriate activities, despite their illness or cognitive capacity. By recognizing and respecting these parental perspectives, HCPs can not only contribute to enhancing the child’s well-being but also support the QoL for parents. This involves acknowledging the importance parents place on their role and tailoring care plans to align with the values and beliefs of each family. However, HCPs should also be careful not to contribute to unrealistically high expectations on parents, as research has shown that interactions with HCPs impact parents’ definitions of what a good parent is [49].

The parents in our study highlighted that the struggle to allocate time and attention to the needs of both the ill child and siblings caused feelings of guilt and inadequacy and affected their QoL. In several studies, parents have identified the focus on siblings as an important but unmet parental need [3, 25, 50]. Parents feel stressed and overwhelmed by the care and attention needed by all of their children [5], as they feel forced to give priority to the needs of the ill child, meaning that siblings often come in second place [3, 32, 51]. Based on our findings, we suggest that practical assistance or respite care for the ill child can enhance parents’ QoL, as it creates the necessary space for parents to engage more fully with siblings.

Our study also revealed that parents’ encounters with professionals in the health and social system affected their QoL. Negative experiences affecting QoL were either due to challenges in the hospital setting, where they felt their competence regarding their own child was overruled, or in municipal settings, where they had to advocate for services due to the limited knowledge of municipal staff related to the comprehensive situation surrounding the ill child’s needs. In the case of the hospital setting, Bogetz’s study shows that many parents in PPC feel unheard and undervalued despite them being experts and advocates for their child’s health and well-being [6]. Other studies have shown that parents value empathy [52], being allowed to speak up for their child [37], and respect for their autonomy and preferences [53]. These findings show that HCPs must create supportive and empathetic healthcare environments in which parents’ competence in relation to their child is acknowledged.

In the case of the municipality setting, our findings are in line with another Norwegian qualitative study [54] in which parents reported that community services lacked professional competence related to the children’s complex needs. A lack of knowledge about PPC is a commonly identified barrier to the provision of palliative care services [52]. Our study found that parents were highly appreciative when professionals from the hospital acted as “translators” of the severe situation to the municipalities. This finding is supported by Rico-Menas’s study, which suggests that promoting training in PPC, prioritizing more horizontal organizations, providing coordination and communication between professionals from different services, and establishing a position of case coordinator could improve understanding in PPC services [55]. The findings show that it is necessary to implement and improve PPC at all levels of health care.

The complexity of elements shaping parents’ QoL is evident. The interconnectedness between parents, the ill child, siblings, and interactions with the health and social care system, highlights the need to understand and address diverse aspects in enhancing parents QoL. Overall, there are interconnected life worlds of the ill child, siblings, and parents, emphasizing the relationship between parents’ QoL and their perceptions of the well-being of both the ill child and the siblings. For instance, the ability to provide their ill child with a good life may improve QoL in one aspect but potentially decrease it in another if time for siblings is compromised. Similarly, while work may contribute to parental QoL, feelings of guilt for not being with the ill child might lead to a decline in QoL. Understanding these dynamics is crucial for HCPs in providing comprehensive support to parents. This involves providing support beyond medical care for the ill child, taking into account the broader family context, as well as strengthening and educating the systems supporting the family in their daily life, such as the municipal health and social services, schools, and kindergartens. This insight aligns with van Manen’s perspective [27], emphasizing that profound knowledge of the studied phenomenon facilitates a practitioner’s ability to be in touch with the situation, understand its meaning and significance, and then act in ways that provide more sensitive and caring professional services. Findings may also guide future research, both qualitative researcher who wants to delve deeper into parents’ QoL, and quantitative researchers focusing on the development of QoL measures for parents.

### Strengths and limitations

Several actions were taken to enhance the trustworthiness of the study [56, 57]. The study’s findings gained credibility through the researcher spending time in the families’ homes, allowing for a comprehensive understanding and deeper insight into the context. Directly asking parents about their QoL, rather than relying solely on interpretations of the material, further enhanced the credibility of the findings. Additionally, the parents’ descriptions were supported by pictures and objects, which contributed to a more comprehensive and nuanced analysis.

The dependability of the research was ensured through the methodological consistency maintained throughout the study, with the same person conducting all interviews and transcriptions. However, a limitation to dependability was van Manen’s open approach to thematic analysis, as this approach does not adhere to specific steps that can be explicitly shown to the reader. Nevertheless, we provided a detailed description with examples illustrating how the analysis was conducted with the goal of enhancing transparency.

To enhance confirmability, the author group, consisting of individuals with expertise in both PPC and qualitative research, continuously reflected on how preunderstandings could potentially influence the research process and shape the emerging findings. Regarding transferability, the extent to which findings can be useful to individuals in other settings [57], descriptions of the context, participants, and analysis were provided so that readers can decide whether the findings are applicable to their settings. The findings may be limited to the experiences of parents of children with cancer and genetic conditions. There may also be differences between these conditions that do not emerge in the study, as our study focused on the overall phenomenon of QoL for parents of children with LT/LL conditions. Another limitation may have been that only parents living in a heterosexual partnership and no single parents were interviewed. Nevertheless, the inclusion of participants with diverse geographic backgrounds and varying levels of education aimed to encompass a range of experiences. The inclusion of both mothers and fathers further strengthened the richness of perspectives.

## Conclusion

The study shows the complex nature of parents’ QoL in the context of living with a child with an LT/LL condition. The interconnection between parents’ QoL, their perceptions of the well-being of both the ill child and siblings, their experience of normality, and interactions with the health and social care system underscores the intricate balance parents must navigate in various aspects of their lives. Understanding these dynamics is crucial for HCPs in providing support that can enhance parents’ QoL in their daily lives.

### Electronic supplementary material

Below is the link to the electronic supplementary material.

Supplementary Material 1

## Data Availability

All questions regarding data can be forwarded to the corresponding author. Due to the purpose limitation in the study approval from the Norwegian Center for Research Data (reference number 289184, art. 5.1 b), we are not allowed to share the transcribed material as individual privacy can be compromised.

## Declarations

### Authors’ information (optional)

All authors are members of the CHIP network. Trine Brun Kittelsen, OTR, MSc, is a PhD candidate at OsloMet with 20 years of experience as a pediatric occupational therapist in habilitation and municipal health services. Vibeke Bruun Lorentsen, PhD, is a nurse and associate professor of the master of clinical nursing, specialization in cancer nursing at VID Specialized University. Anja Lee, PhD, MD, has been a pediatrician for many years and is currently leading the first Norwegian PPC team established in 2019 at Oslo University Hospital. Lisbeth Gravdal Kvarme, PhD, is a professor of public health nursing at OsloMet with clinical experience in a hospital for severely ill children. Charlotte Castor, PhD, is an associate senior lecturer at Lunds University with 25 years of clinical nursing experience and is a researcher within home care services for ill children and PPC. Anette Winger, PhD, is a professor in palliative care for children and adolescents at OsloMet. She is an experienced pediatric nurse, educator, and researcher in pediatric palliative care.

## Abbreviations

PPC: Pediatric palliative care
QoL: Quality of Life
LT/LL: Life-threatening or life-limiting
CHIP: Children in Palliative Care
TfSL: Together for Short Lives
HCP: Health care professional
