# Supplementary material for: It’s about living a normal life: parents’ quality of life when their child has a life-threatening or life-limiting condition - a qualitative study
Source: BMC Palliat Care. 2024 Apr 9;23:92. doi: 10.1186/s12904-024-01417-3 (PMC11003040; doi:10.1186/s12904-024-01417-3)
Supplement: Supplementary file 1 — Supplementary Material 1 [file 12904_2024_1417_MOESM1_ESM.docx]

**SUPPLEMENTARY FILE**

**INTERVIEW GUIDE - PARENTS**

**For the interviewer**

- Before the interview, adopting a curious, present, and open attitude is important.

**Information for parents before the interview**

- "I would like to hear about your experiences *living* with a child with an LT/LL condition. I am interested to hear about what you perceive as QoL and what you perceive as help and challenges in daily life."
- "You are the experts, and there's nothing 'wrong' to talk about."
- "I'll start by asking you to share information about the child and your situation. To ensure you have the opportunity to discuss what's important to you, I'll let you lead the interview, while providing follow-up questions and introducing additional queries along the way."
- "Feel free to discuss the object or picture you've brought along whenever it suits you."
- "While my education is in occupational therapy, my current role is that of a researcher. This means my focus is on your experiences rather than topics typically of interest to occupational therapists. Nevertheless, my background allows me to grasp and comprehend various medical terms."
- "Please be aware that you may experience emotional reactions after talking about your situation. I'll reach out to you after a week to hear how you are doing. "

| **Questions**   - "Can you please tell me about your child?" - "Can you please tell me about a good day?" - "Can you please tell me about a bad day?" - "What is the difference between a good day and a bad day?" - "Now, I will ask a question about QoL. What is QoL when living with a child with an LT/LL condition?" - "What do you experience as challenges in daily life?" - "What do you experience as help in daily life?" - "Is there something particularly important that you've shared and want to ensure I fully understand?"   **Follow-up questions**   - "Could you give an example?" - "Could you recall a specific time when that happened?" - "Could you tell me more?" - "Could you describe how…" - "What happened after…?" - "In which way did you…?" - "What is it that…?" - "How was it to…?" - "How does it feel to…?" - "Who was there then?" - "Could you tell me the location or place where it happened?"   **Follow-up questions when parents show a thing or picture**   - “Can you please tell me what’s on the picture? Please say it for recording on the recorder." - “Can you tell me why the thing /picture is important?” - Reflect together around the answer |
| --- |
